# Using Synthetic Health Care Data to Leverage Large Language Models for Named Entity Recognition: Development and Validation Study

**DOI:** 10.2196/66279

**Published:** 2025-03-18

**Authors:** Hendrik Šuvalov, Mihkel Lepson, Veronika Kukk, Maria Malk, Neeme Ilves, Hele-Andra Kuulmets, Raivo Kolde

**Affiliations:** 1 Institute of Computer Science University of Tartu Tartu Estonia

**Keywords:** natural language processing, named entity recognition, large language model, synthetic data, LLM, NLP, machine learning, artificial intelligence, language model, NER, medical entity, Estonian, health care data, annotated data, data annotation, clinical decision support, data mining

## Abstract

**Background:**

Named entity recognition (NER) plays a vital role in extracting critical medical entities from health care records, facilitating applications such as clinical decision support and data mining. Developing robust NER models for low-resource languages, such as Estonian, remains a challenge due to the scarcity of annotated data and domain-specific pretrained models. Large language models (LLMs) have proven to be promising in understanding text from any language or domain.

**Objective:**

This study addresses the development of medical NER models for low-resource languages, specifically Estonian. We propose a novel approach by generating synthetic health care data and using LLMs to annotate them. These synthetic data are then used to train a high-performing NER model, which is applied to real-world medical texts, preserving patient data privacy.

**Methods:**

Our approach to overcoming the shortage of annotated Estonian health care texts involves a three-step pipeline: (1) synthetic health care data are generated using a locally trained GPT-2 model on Estonian medical records, (2) the synthetic data are annotated with LLMs, specifically GPT-3.5-Turbo and GPT-4, and (3) the annotated synthetic data are then used to fine-tune an NER model, which is later tested on real-world medical data. This paper compares the performance of different prompts; assesses the impact of GPT-3.5-Turbo, GPT-4, and a local LLM; and explores the relationship between the amount of annotated synthetic data and model performance.

**Results:**

The proposed methodology demonstrates significant potential in extracting named entities from real-world medical texts. Our top-performing setup achieved an *F*_1_-score of 0.69 for drug extraction and 0.38 for procedure extraction. These results indicate a strong performance in recognizing certain entity types while highlighting the complexity of extracting procedures.

**Conclusions:**

This paper demonstrates a successful approach to leveraging LLMs for training NER models using synthetic data, effectively preserving patient privacy. By avoiding reliance on human-annotated data, our method shows promise in developing models for low-resource languages, such as Estonian. Future work will focus on refining the synthetic data generation and expanding the method’s applicability to other domains and languages.

## Introduction

In the era of data-driven health care, the effective extraction of valuable information from electronic health records (EHRs) has become increasingly vital for informed decision-making, medical research, and public health initiatives. Named entity recognition (NER) stands as a cornerstone in the realm of natural language processing, playing a pivotal role in the automated extraction of named entities, such as diseases, medications, symptoms, and medical procedures, from unstructured text [1]. NER facilitates the transformation of text data into structured information, enabling health care professionals and researchers to carry out statistical analyses on the data.

While NER has achieved remarkable success in several languages and domains, the journey to attain proficient NER models for low-resource languages presents intricate challenges [1-3]. Estonian, a Finno-Ugric language spoken by approximately 1.1 million people, is one such language that grapples with limited linguistic resources, especially in specialized domains such as health care, largely because health records are sensitive, personalized data, which makes sharing them for labeling very difficult [4] in addition to the task being already time-consuming and expensive. The same challenges are faced with storing and sharing the annotated data. To tackle this, attempts have been made at removing or masking personally identifiable information [5,6]; however, this can still leave the data vulnerable to reconstruction attacks [6]. Despite its importance, the scarcity of annotated Estonian health care text data has hindered the development of robust NER models tailored to the unique linguistic features and domain-specific nuances of the language. At the same time, large language models (LLMs) such as GPT-4 have been shown to provide impressive results in tasks related to understanding different languages [7].

Many approaches using transformer-based models have been applied to EHRs to extract medical named entities [8-10]; however, these approaches primarily rely on human-annotated data for the training data, which we do not use for training. Most transformer-based approaches rely on using a model pretrained on a large corpus of text and fine-tuning it with the annotated data.

To tackle the problem of low-resource languages lacking annotation data, attempts have been made to improve the model using data from other languages via transfer learning [11,12], which has been shown to improve the model’s performance by adding resources from languages with more data.

With the rapid increase in the performance of LLMs, attempts have also been made to leverage these models to extract named entities from texts. It has been shown that using GPT-3 for annotating the texts can be much cheaper and faster than using humans; however, there is room for improvements in the quality of the annotations made by the model [13].

Tang et al [14] used ChatGPT (OpenAI) [15] to generate synthetic medical texts with annotations to train a downstream model for different applications including NER. The performance of the resulting models was promising, considering the approach of using synthetic data. Their approach uses ChatGPT directly to generate the synthetic texts, meaning there is no potential for possible data leakage from the synthetic texts. However, because the GPT models were trained on data that are publicly available, they are not trained to understand the nuances of a specific medical domain in a low-resource language, and the synthetic data can be very different from real EHRs, possibly causing poor performance for the downstream model.

This study introduces a comprehensive pipeline using LLMs to train an NER model, enabling the annotation of targeted entities within health care texts. First, synthetic Estonian EHRs are generated by GPT-2 [16] trained locally on real data. We generate the synthetic data to avoid sharing sensitive information with third-party applications. These are passed to GPT-3.5-Turbo and GPT-4 as payload in a prompt, instructing the model to annotate the text with entities specified by the user. The responses are parsed and converted into training data for token classification with XLM-RoBERTa [17], which can be used on the downstream task of annotating real data. This approach ensures that sensitive data do not get passed to third-party LLM hosts via their application programming interface (API) calls and enables all the training to be carried out on-site.

We showcase the differences in performance between the selected prompts. We then compare the performance of GPT-3.5-Turbo, GPT-4, and a local Llama-based model for the best prompt. We continue with using the best prompt with GPT-4 to annotate generated synthetic data in batches, showcasing the improvement of the model based on the amount of training data. Finally, we discuss the performance of this method and possible room for improvements and conclude the results of this paper.

With this paper, we aim to show how we take advantage of powerful LLMs for training locally usable NER models without risking the privacy of patient records by using synthetically generated data.

## Methods

### Study Design

**Figure 1:**
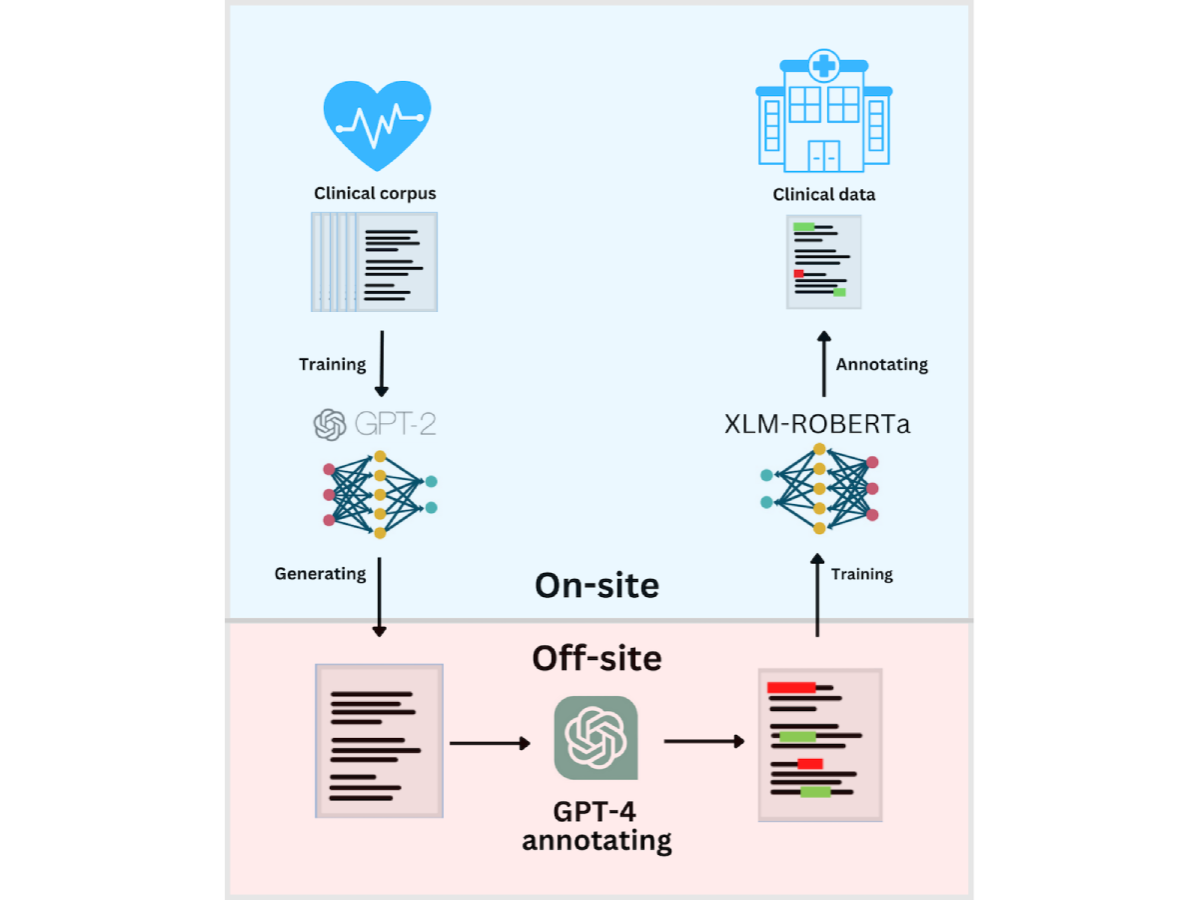
Illustration of pipeline.

Our work follows the pipeline depicted in Figure 1. OpenAI models were selected for annotating the synthetic data due to their availability and their existing wide use in research. The clinical corpus was used for training the GPT-2 model, which we used to generate synthetic data. We then passed the synthetic data to GPT-4 through an API, asking it to annotate the data. The results were parsed and converted into training data for a downstream model, which we used to annotate real-world data.

### Generating Synthetic Data With GPT-2

As health care data are very sensitive, sharing them with third-party LLM hosts such as OpenAI or Azure (Microsoft Corp) is not possible. For this reason, we used synthetic data for the annotating process, generated by a GPT-2 small model. The dataset we used for training the model consists of around 10 million texts from 200,000 Estonians collected between 2010 and 2020 [18]. The texts are from a national registry of discharge reports covering both primary and secondary care contacts. The documents include anamnesis, summary, test results, objective findings, and procedures. During training, each medical document was prefaced with the document type, patient’s age group, patient’s gender, and diagnosis code, a similar idea to that used by Keskar et al [19], to generate synthetic health care records based on our specifications.

As the model can generate exact copies of the text it was trained on, it is important to ensure that no generated texts are identical to the training data. One way to verify this is by comparing the generated text directly against all training data. However, this method is unreliable because it fails to detect copies when even a single token differs. Some works [20,21] check if n-grams with a length of 13 in the generated texts are present in the training data, and if there is a match, then the generated texts are considered unoriginal. The problem with such an approach is that it can consider the training text with a length of 13 originals if we switch 1 word in it with a synonym. To address this, we considered using the longest common subsequence (LCS). For each generated text, we calculated the LCS with each training text, selected the longest, and divided by the generated text length, producing a score between 0 (unique) and 1 (identical).



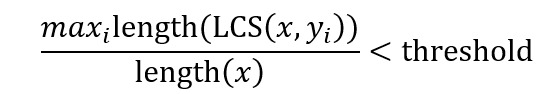



The advantage of LCS is its ability to classify a generated text as a copy of the training data, even if some words have been replaced, deleted, or added. While calculating the LCS for every training instance is computationally expensive, with a training dataset of 10 million texts, we felt it was feasible and critical to ensure that no training text (real medical texts) copies were present in subsequent steps. However, with longer texts, the LCS elements were scattered across hundreds of tokens in the training text not representing continuous expressions. To mitigate this issue, we explored using a penalized LCS, which factors in the distances between matched tokens within the sequence. Given a penalty length *k*, we subtracted a penalty term *distance/k* for each consecutive LCS element, where the distance between LCS elements is the maximum of distances in *x* and *y_i_*. If the distance is larger than *k*, we would subtract 1. If *k* is 1, then this is the same as finding the longest n-gram. For larger *k* values, the penalty would be smaller and we would match more texts. We experimented with various penalties and found 20 to be effective in removing scattered matches without being overly restrictive.

### Using LLMs for Annotations

The synthetic texts generated by our custom GPT-2 were passed as payloads into a prompt for Azure Cognitive Services GPT models via their API, which were instructed to annotate the data. We opted out of human review of data in Azure to further ensure the privacy of even the synthetic EHRs. For all experiments, we use the default parameters for the models. Our goal was to find the corresponding text spans for each annotation class; however, LLMs have been shown to make errors when prompted to return indexes for text spans [22], and our experiments showed that both the GPT-3.5-Turbo and GPT-4 models gave us incorrect spans when asked. Therefore, we only asked it to give us the text and the annotation class and map it to the text, finding the spans manually. To make the results as easy to parse as possible, we asked it to return the results in a *JSON* format, with each annotation class as an element and each text as a value of the corresponding element.

Prompt engineering has been shown to largely influence the quality of the output of the models [23]. This led us to select 4 different prompts to test how well they performed so we could choose the best one to annotate the data with. The prompts are listed in Table 1.

First, we used a zero-shot prompt because it is the most simplistic way to ask a model to complete a task. Our zero-shot prompt includes a description of the task, the wanted output format, and the input text.

The second prompt consists of instructions in Estonian to test if the accuracy of the annotations improves if the language of the prompt matches the language of the data. It is the zero-shot prompt translated into Estonian.

As human annotators have annotation guidelines as their instructions, we decided to explore whether adding definitions of entities in the prompts would increase the accuracy of the annotations. By providing definitions, we can specify what counts as an entity in our domain rather than relying on the model’s understanding of the terms. The definitions for the classes were written by the same medical expert who annotated the synthetic data against which we tested the accuracy of the GPT model’s annotations. Our prompt with definitions was inspired by Li et al [24] because they had promising results using prompts with additional annotation guidelines for NER with a BERT model.

As GPT models have been shown to perform better when provided with examples of task completion [20], we also tried a few-shot prompt. It consists of 5 pairs of example inputs and the expected *JSON* outputs. Few-shot prompts are particularly effective in low-resource settings, as they provide semantically relevant context that helps bridge the language gap between the target language and the high-resource language the model is proficient in [23].

The resulting responses were parsed in Python (Python Software Foundation) to convert them into *.tsv* format for training the downstream model. It was carried out by searching for the annotation entities from the original text, mapping them to spans, and finally aligning the spans into tokens.

**Table 1:** Contents of prompts.

Prompt name	Prompt
Zero-shot	In the text below, give the list of: drug named entity, procedure named entity, disease named entity, smoking named entity. Words need to be in exactly the same format as in input text. Format the output in JSON with the following keys: DRUG for drug named entity, PROCEDURE for procedure named entity, DISEASE for disease named entity, SMOKING for smoking named entity. Text below:
Estonian	Loetle järgnevas tekstis järgmiseid nimeolemeid: ravimite nimeolemid, protseduuride nimeolemid, haiguste nimeolemid, suitsetamiste nimeolemid. Sõnad päevad olema täpselt samal kujul kui sisendtekstis. Vorminda väljund JSON kujul järgnevate võtmetega: RAVIM ravimite nimeolemite jaoks, PROTSEDUUR protseduuride nimeolemite jaoks, HAIGUS haiguste nimeolemite jaoks, SUITS suitsetamiste nimeolemite jaoks. Tekst on järgmine:
Definitions	In the text below, give the list of drug named entity, procedure named entity, disease named entity, smoking named entity. Words need to be in exactly the same format as in input text. The annotation guidelines are the following: DRUG - any names or active ingredients of drugs, including abbreviations; no quantities PROCEDURE - any mentions of procedures, analyses etc, including also cites (e.g. “ajupiirkonna mrt”); no single blood analyses, only overall mentions of blood analyses DISEASE - names of diseases, ICD-10 codes SMOKING - any mentions of smoking, smoking status, both negative and positive Format the output in JSON with the following keys: DRUG for drug named entity, PROCEDURE for procedure named entity, DISEASE for disease named entity, SMOKING for smoking named entity. Text below:
Few-shot	In the text below, give the list of: drug named entity, procedure named entity, disease named entity, smoking named entity. Words need to be in exactly the same format as in input text. Format the output in JSON with the following keys: DRUG for drug named entity, PROCEDURE for procedure named entity, DISEASE for disease named entity, SMOKING for smoking named entity. Here are a few examples: <Example input 1> <Example output 1> <Example input 5> <Example output 5> Text below:

### Evaluating the Performance of the GPT Models’ Annotations

To evaluate which prompt to use for annotating the data, we generated 500 synthetic texts with 5 of the most frequent *International Classification of Diseases, Tenth Revision* (*ICD-10*) codes in our dataset. For each code, we generated 67 patient summaries and 33 procedures ranging from all age groups and both genders. This dataset was then annotated by a medical expert for drugs, procedures, diseases, and smoking entities to compare the LLM annotations with human annotations. The numbers of entities for each class are depicted in Table 2.

We compared the annotations made by the LLM word level by taking the human annotations as the ground truth and measuring precision, recall, and *F*_1_-score (harmonic mean of precision and recall) of the annotations. We do this using the *seqeval* [25] library to obtain the classification reports with the default options from the annotations.

After determining the most effective prompt for all the annotation classes, we used the prompt with 3 different models—GPT-3.5-Turbo; GPT-4; and Llammas [26], a Llama-2-7B–based [27], instruction-tuned model adapted to Estonian.

This gave us an estimation of how well the different models annotate the data and which prompt to use for annotating the data that are used for training the downstream model.

**Table 2:** Entity counts.

Annotation class	Entity count, n
Drug	219
Procedure	643
Smoking	58
Disease	322

### Training Downstream XLM-RoBERTa Model

Once the responses from the GPT models were parsed into training data format, we used this as data for fine-tuning a BERT-based model. We chose the XLM-RoBERTa base with 125M parameters as its pretraining data includes Estonian and other languages similar to Estonian. Additionally, because it is multilingual, we could later add annotations from other languages to further improve its performance. For each model we trained, we used 15% of the training data for the validation set.

The parameters for fine-tuning the model were the following:

- batch_size=16
- evaluation_strategy=“epoch”
- learning_rate=“2e-5”
- per_device_train_batch_size=16
- per_device_eval_batch_size=16
- num_train_epochs=8
- weight_decay=0.01

All the trained models were evaluated at the token level with *seqeval* default settings using 300 documents from real-world data as the test set. We chose 150 texts from patient medical histories and 150 texts from patient procedure texts to make sure we had enough test data for all the annotation classes. We had 2 medical experts annotate these texts for drugs and procedures. The instructions given to the annotators were only the names of the entity classes to ensure that the instructions were similar to the ones given to the models annotating the data. We used one set of annotations as ground truth for our experiments and the second set to calculate the pairwise interannotator agreement [28], which was an *F*_1_-score of 0.73 for drugs and 0.48 for procedures. Once the models were trained on the synthetic data annotated by the GPT models, we could use the models on the test data in a secure local environment and retrieve the results.

### Ethical Considerations

The medical texts were accessed according to the Estonian Committee on Bioethics and Human Research approval (1.1-12/3797) that waived the requirement of informed consent for the use of the data. The methodology presented in this study prioritizes patient privacy by exclusively using synthetic health care data for model training instead of augmenting original data. This means there is no direct link between the patients and the generated texts. We furthermore ensured privacy by removing verbatim and close-to-verbatim copies of synthetic texts. All sensitive medical records remain protected, and synthetic data were generated using local models. This ensures that no actual patient data are shared with external platforms, in compliance with privacy laws and ethical standards governing medical data use.

## Results

### Overview

To test the performance of the pipeline as a whole, we chose 4 annotation classes that we wanted to extract from the texts—drugs, procedures, smoking, and disease. Extracting drugs and smoking is a simple task with relatively fixed vocabularies that we hypothesized would be easy to learn for the model. Procedures and diseases are slightly trickier, as they are often written in shorthand and their representation may vary, making context very important.

### Generating the Data

Based on the annotation classes for the experiment, we chose procedures and patient medical history for the types of EHRs we wished to generate. We also added specifications for 13 age groups, ranging from 15 to 80 years, to generate data for more diverse patients. We chose C50 as the *ICD-10* diagnosis code, generating the texts for patients with malignant neoplasms of the breast. We started with 10,000 generated texts and used our LCS algorithm to filter out texts that were too close to the original data. We ended up with around 4100 documents we could use for annotating with LLMs, out of which 2700 were of medical history and 1400 of procedures.

### Prompt Selection

We used all the selected prompts for annotating the 500 synthetically generated texts with GPT-3.5-Turbo to compare their performance. For each prompt, we compared the annotations made by the GPT-3.5-Turbo model with the human annotations. The results for each prompt are depicted in Table 3. For precision and recall, 95% CIs were calculated using the Wilson score method [29]. The parsed responses column displays how many of the responses contained a valid *JSON* element containing the annotation classes.

For drugs, procedures, and smoking, the highest *F*_1_-score was reached by using the few-shot prompt on GPT-3.5-Turbo. In the case of diseases, using the definitions prompt resulted in the highest *F*_1_-score of 0.44, although the Estonian prompt had a higher recall of 0.52 versus 0.43. For smoking, the definitions prompt gave the highest precision of 1 versus 0.92 for the few-shot prompt; however, its recall and *F*_1_-score were lower than those of the few-shot prompt.

We selected the prompt with the highest combined *F*_1_-score for all the classes, which was the few-shot prompt, to continue annotating the rest of the synthetic data. We first used the same training data that GPT-3.5-Turbo annotated and redid it both with GPT-4 and a locally hosted Llammas model to compare the differences in performance. The results are depicted in Table 4.

In all instances, GPT-4 had a higher *F*_1_-score than the GPT-3.5-Turbo and Llammas models; however, for procedure and smoking, GPT-3.5-Turbo had the highest precision.

**Table 3:** GPT-3.5-Turbo annotation performance compared to human expert annotations.

Annotation class and prompt		Precision (95% CI)	Recall (95% CI)	*F*_1_-score	Parsed responses, n
**Drug**					
	Prompt 1: zero-shot	0.61 (0.55-0.67)	0.75 (0.69-0.80)	0.67	495
	Prompt 2: Estonian	0.3 (0.26-0.34)	0.63 (0.56-0.69)	0.41	487
	Prompt 3: definitions	0.6 (0.54-0.65)	0.82 (0.76-0.87)	0.7	500
	Prompt 4: few-shot	0.63 (0.57-0.68)	0.88 (0.83-0.92)	0.74	495
**Procedure**					
	Prompt 1: zero-shot	0.25 (0.21-0.3)	0.15 (0.12-0.18)	0.19	495
	Prompt 2: Estonian	0.24 (0.21-0.28)	0.22 (0.19-0.25)	0.23	487
	Prompt 3: definitions	0.25 (0.22-0.28)	0.26 (0.23-0.3)	0.26	500
	Prompt 4: few-shot	0.34 (0.3-0.38)	0.26 (0.23-0.3)	0.3	495
**Smoking**					
	Prompt 1: zero-shot	1 (0.65-1)	0.12 (0.06-0.23)	0.22	495
	Prompt 2: Estonian	0.21 (0.14-0.31)	0.29 (0.19-0.42)	0.24	487
	Prompt 3: definitions	1 (0.85-1)	0.36 (0.25-0.49)	0.53	500
	Prompt 4: few-shot	0.92 (0.75-0.98)	0.41 (0.29-0.54)	0.57	495
**Disease**					
	Prompt 1: zero-shot	0.24 (0.21-0.27)	0.5 (0.45-0.55)	0.32	495
	Prompt 2: Estonian	0.12 (0.1-0.14)	0.52 (0.47-0.57)	0.2	487
	Prompt 3: definitions	0.44 (0.39-0.5)	0.43 (0.38-0.48)	0.44	500
	Prompt 4: few-shot	0.4 (0.35-0.45)	0.5 (0.45-0.55)	0.44	495

**Table 4:** Few-shot annotation performance of different models compared to human annotations.

Annotation class and model			Precision (95% CI)		Recall (95% CI)		*F*_1_-score
**Drug**							
	GPT-3.5-Turbo	0.63 (0.57-0.68)		0.88 (0.83-0.92)		0.74	
	GPT-4	0.66 (0.6-0.71)		0.88 (0.83-0.92)		0.75	
	Llammas	0.63 (0.57-0.69)		0.67 (0.61-0.73)		0.65	
**Procedure**							
	GPT-3.5-Turbo	0.34 (0.3-0.38)		0.26 (0.23-0.3)		0.3	
	GPT-4	0.33 (0.3-0.36)		0.42 (0.38-0.46)		0.37	
	Llammas	0.28 (0.24-0.32)		0.22 (0.19-0.25)		0.25	
**Smoking**							
	GPT-3.5-Turbo	0.92 (0.75-0.98)		0.41 (0.29-0.54)		0.57	
	GPT-4	0.69 (0.55-0.8)		0.55 (0.42-0.67)		0.61	
	Llammas	0.75 (0.47-0.91)		0.16 (0.09-0.27)		0.26	
**Disease**							
	GPT-3.5-Turbo	0.4 (0.35-0.45)		0.5 (0.45-0.55)		0.44	
	GPT-4	0.4 (0.36-0.44)		0.7 (0.65-0.75)		0.51	
	Llammas	0.21 (0.18-0.25)		0.35 (0.3-0.4)		0.26	

### Training the Downstream Model

We used the 2700 medical history texts and 1400 procedure texts resulting from LCS filtering to create batches of training data to see how well the model performs with additional training data. For each batch, we selected two-thirds of the texts from medical history and one-third from procedures, to fully use all the training data. Each model was then validated on 300 real-world documents annotated by a medical expert for drugs and procedures.

**Figure 2:**
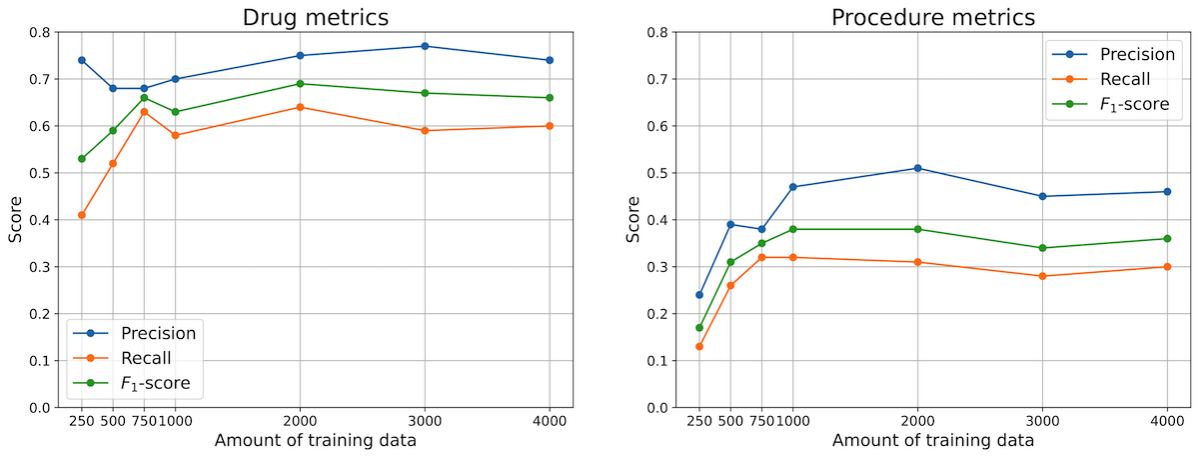
Annotation scores on validation data (N=300) for models of different amounts of training data.

Figure 2 shows the performance of the model on the drug and procedure annotation classes. For every amount of data depicted on the x-axis, a downstream model was trained and evaluated on the test data.

For drugs, the performance of the model increased until 750 texts, lowered slightly for 1000 texts, and reached the peak at 2000 texts, achieving a precision of 0.75, a recall of 0.64, and an *F*_1_-score of 0.69. In the case of procedures, the model peaked at 1000 synthetic annotated texts, achieving a precision of 0.47, a recall of 0.32, and an *F*_1_-score of 0.38. For 2000 texts, the *F*_1_-score remained the same; however, the precision increased by 0.04 whereas the recall dropped by 0.01.

We have used the annotations made by the second medical expert as validation data to show how the metrics vary with annotation variability. The results showed similar dynamics to the scores shown in Figure 2, although the absolute *F*_1_-scores were slightly lower for procedures (Multimedia Appendix 1).

## Discussion

### Principal Findings

For the final model trained on different batches of data with annotations made by GPT-4, we found that the best-performing model achieved an *F*_1_-score of 0.69 for the drug and an *F*_1_-score of 0.38 for procedures. The result was achieved with 2000 annotated synthetic texts for drugs and 1000 for procedures. Training the models on more texts did not improve the results. Based on these results, it seems there is a cap on how much one can gain from synthetic data using this method. The downstream model seems to learn the task after 2000 texts, and afterward, adding more data just creates more noise and possibly makes the model overfit on similar synthetic texts.

It is important to note that the interannotator agreement showed an *F*_1_-score of 0.73 for drugs and 0.48 for procedures, which means there is inherently a lot of room for disagreement and subjectivity between the entities. In many instances, the disagreement stemmed from how many prefixes to include for the entity (eg, location of the procedure). This result tells us that achieving a perfect *F*_1_-score is not realistic, as ground truth itself can be subjective and depend on the annotators, further strengthening the achieved result of this paper. The disagreement between the medical experts and the LLM can be somewhat mitigated by providing more clear annotation guidelines for both the medical experts and the LLMs annotating the data.

We found that the most effective prompt for generating accurate annotations for our downstream model was the few-shot approach. Notably, while the texts themselves were in Estonian, prompts provided in English consistently produced more precise annotations compared to prompts written in Estonian. Openly available, multilingual LLMs are known to be heavily trained on English text, as English is vastly overrepresented in publicly available datasets. As a result, the model’s understanding of English instructions and its ability to follow them are significantly more robust than for low-resource languages such as Estonian [30,31]. The local Llammas model did not perform as well as the GPT-3.5-Turbo, likely because it is a much smaller model, and parsed only 454 of the responses, meaning in some instances it did not return coherent outputs.

We then found that when comparing the performance of the annotations between GPT-3.5-Turbo, GPT-4, and Llammas, GPT-4 performed better per *F*_1_-score for all cases, which was to be expected, because it is a larger and more powerful model. For procedures and smoking, the GPT-3.5-Turbo model achieved a higher precision; however, in the case of procedure, it was only by 0.01, and in the case of smoking, even though the difference was 0.23, there were only 58 entities in the texts, meaning the difference in results is not very reliable.

### Comparison to Prior Work

While our model’s results do not surpass standard transformer-based models trained on human-annotated data, which achieve *F*_1_-scores of 0.9 for drugs [8,9] and 0.7 for procedures [8], it is crucial to contextualize this comparison. These prior results are derived from English, a high-resource language with abundant training data. In contrast, our approach demonstrates the efficacy of using synthetically generated data for low-resource languages such as Estonian, without relying on human annotations. Considering that our approach does not use any human-annotated data and the models are all trained on synthetically generated data, a good starting point is to use synthetic data for an initial model that can be used to enrich a model trained on real data or help annotators with their tasks on real-world data.

### Future Directions

The pipeline of the method described in this paper consists of many pieces, each with room for improvement. First, both the generation and annotation of synthetic data could be enhanced by leveraging more advanced models. The synthetic texts were generated by GPT-2, but newer model architectures with more parameters could outperform this and generate better data, while more specialized models for clinical texts, such as Me-LLaMA [32] and GatorTron [33], could improve annotation accuracy. Second, we saw a noticeable change in performance depending on the prompt used, suggesting that further prompt engineering could lead to more improvements. Third, human annotators in health care settings typically rely on lengthy annotation guidelines, while in contrast, our method provided only 5 examples. A more detailed description of annotation classes could improve the accuracy of model-generated annotations.

### Conclusion

In this paper, we took advantage of powerful LLMs GPT-3.5-Turbo and GPT-4 without risking the privacy of the data by using synthetically generated data. We used these models to annotate the synthetic texts, on which we locally trained a BERT-based NER model for use on real-world data. We obtained an *F*_1_-score of 0.69 for drugs and an *F*_1_-score of 0.38 for procedures, which are impressive, given that the data used were synthetic and without human annotations. We then showcased the difference in performance using GPT-3.5-Turbo and GPT-4 for the task, finding that GPT-4 is substantially better for the task. We also showed how the performance of the model is influenced by the amount of synthetic training data used for training it. Altogether, the proposed method allows for developing NER models while removing the bottleneck of using medical experts for data annotation, automating the process of extracting facts from unstructured data while preserving patient privacy.

## Appendix

Multimedia Appendix 1 Annotation scores on validation data (N=300) from the second medical expert for models of different amounts of training data.

## Abbreviations

API: application programming interface
EHR: electronic health record
ICD-10: International Classification of Diseases, Tenth Revision
LCS: longest common subsequence
LLM: large language model
NER: named entity recognition

## Data Availability

The medical texts from the Estonian Biobank were used according to the Estonian Committee on Bioethics and Human Research approval (1.1-12/3797). These data cannot be shared publicly because they may contain identifiable information about the participants. To access these data, official procedures outlined in reference [34] have to be followed. The synthetic data used for annotations can be accessed on a case-by-case basis upon contacting author RK. The code for the pipeline is publicly available in GitHub [35].
