# Supplementary material for: Using Synthetic Health Care Data to Leverage Large Language Models for Named Entity Recognition: Development and Validation Study
Source: J Med Internet Res. 2025 Mar 18;27:e66279. doi: 10.2196/66279 (PMC11962312; doi:10.2196/66279)
Supplement: Multimedia Appendix 1 [file jmir_v27i1e66279_app1.docx]

**Multimedia Appendix 1.** Annotation scores on validation data (N=300) from the second medical expert for models of different amounts of training data.
